# Supplementary material for: Unveiling the Bioactive Potential of the Invasive Jellyfish Phyllorhiza punctata Through Integrative Transcriptomic and Proteomic Analyses
Source: Biomolecules. 2025 Aug 4;15(8):1121. doi: 10.3390/biom15081121 (PMC12383608; doi:10.3390/biom15081121)
Supplement: Supplementary file 1 [file biomolecules-15-01121-s001.zip › Suplementary Material/FileS3/PEPFOLD4/XP_065058319_14272/clusters.html]

 PEP-FOLD clusters


Download archive of all models  here   

|  |  |  |
| --- | --- | --- |
| # | file | sOPEP |
| # cluster 1 - Size: 3 |  |  |
|  | XP\_065058319\_14272-model1 | -28.2844 |
|  | XP\_065058319\_14272-model1.1 | -26.6795 |
|  | XP\_065058319\_14272-model1.2 | -24.069 |
| # cluster 2 - Size: 10 |  |  |
|  | XP\_065058319\_14272-model2 | -26.9143 |
|  | XP\_065058319\_14272-model2.1 | -26.2248 |
|  | XP\_065058319\_14272-model2.2 | -26.2103 |
|  | XP\_065058319\_14272-model2.3 | -25.9845 |
|  | XP\_065058319\_14272-model2.4 | -25.9799 |
|  | XP\_065058319\_14272-model2.5 | -25.9303 |
|  | XP\_065058319\_14272-model2.6 | -25.6879 |
|  | XP\_065058319\_14272-model2.7 | -25.458 |
|  | XP\_065058319\_14272-model2.8 | -24.0361 |
|  | XP\_065058319\_14272-model2.9 | -24.0225 |
| # cluster 3 - Size: 3 |  |  |
|  | XP\_065058319\_14272-model3 | -26.4256 |
|  | XP\_065058319\_14272-model3.1 | -26.0622 |
|  | XP\_065058319\_14272-model3.2 | -25.6533 |
| # cluster 4 - Size: 6 |  |  |
|  | XP\_065058319\_14272-model4 | -25.89 |
|  | XP\_065058319\_14272-model4.1 | -25.5332 |
|  | XP\_065058319\_14272-model4.2 | -24.963 |
|  | XP\_065058319\_14272-model4.3 | -24.6547 |
|  | XP\_065058319\_14272-model4.4 | -24.4346 |
|  | XP\_065058319\_14272-model4.5 | -23.87 |
| # cluster 5 - Size: 3 |  |  |
|  | XP\_065058319\_14272-model5 | -25.8836 |
|  | XP\_065058319\_14272-model5.1 | -25.4878 |
|  | XP\_065058319\_14272-model5.2 | -23.6864 |
| # cluster 6 - Size: 3 |  |  |
|  | XP\_065058319\_14272-model6 | -25.7037 |
|  | XP\_065058319\_14272-model6.1 | -25.2802 |
|  | XP\_065058319\_14272-model6.2 | -24.6829 |
| # cluster 7 - Size: 2 |  |  |
|  | XP\_065058319\_14272-model7 | -25.2621 |
|  | XP\_065058319\_14272-model7.1 | -25.0446 |
| # cluster 8 - Size: 1 |  |  |
|  | XP\_065058319\_14272-model8 | -25.128 |
| # cluster 9 - Size: 1 |  |  |
|  | XP\_065058319\_14272-model9 | -24.9984 |
| # cluster 10 - Size: 3 |  |  |
|  | XP\_065058319\_14272-model10 | -24.76 |
|  | XP\_065058319\_14272-model10.1 | -22.1741 |
|  | XP\_065058319\_14272-model10.2 | -22.1379 |
| # cluster 11 - Size: 3 |  |  |
|  | XP\_065058319\_14272-model11 | -24.3595 |
|  | XP\_065058319\_14272-model11.1 | -24.0966 |
|  | XP\_065058319\_14272-model11.2 | -21.5633 |
| # cluster 12 - Size: 1 |  |  |
|  | XP\_065058319\_14272-model12 | -23.8997 |
| # cluster 13 - Size: 1 |  |  |
|  | XP\_065058319\_14272-model13 | -23.8808 |
| # cluster 14 - Size: 2 |  |  |
|  | XP\_065058319\_14272-model14 | -23.8752 |
|  | XP\_065058319\_14272-model14.1 | -22.6915 |
| # cluster 15 - Size: 1 |  |  |
|  | XP\_065058319\_14272-model15 | -23.0848 |
| # cluster 16 - Size: 1 |  |  |
|  | XP\_065058319\_14272-model16 | -23.0228 |
| # cluster 17 - Size: 3 |  |  |
|  | XP\_065058319\_14272-model17 | -22.9688 |
|  | XP\_065058319\_14272-model17.1 | -22.9291 |
|  | XP\_065058319\_14272-model17.2 | -20.8486 |
| # cluster 18 - Size: 1 |  |  |
|  | XP\_065058319\_14272-model18 | -22.7033 |
| # cluster 19 - Size: 2 |  |  |
|  | XP\_065058319\_14272-model19 | -22.4019 |
|  | XP\_065058319\_14272-model19.1 | -18.3933 |
| # cluster 20 - Size: 4 |  |  |
|  | XP\_065058319\_14272-model20 | -22.347 |
|  | XP\_065058319\_14272-model20.1 | -20.3686 |
|  | XP\_065058319\_14272-model20.2 | -18.2105 |
|  | XP\_065058319\_14272-model20.3 | -16.3782 |
| # cluster 21 - Size: 3 |  |  |
|  | XP\_065058319\_14272-model21 | -22.326 |
|  | XP\_065058319\_14272-model21.1 | -21.2668 |
|  | XP\_065058319\_14272-model21.2 | -20.9955 |
| # cluster 22 - Size: 3 |  |  |
|  | XP\_065058319\_14272-model22 | -22.2764 |
|  | XP\_065058319\_14272-model22.1 | -22.1563 |
|  | XP\_065058319\_14272-model22.2 | -21.4932 |
| # cluster 23 - Size: 1 |  |  |
|  | XP\_065058319\_14272-model23 | -22.1107 |
| # cluster 24 - Size: 1 |  |  |
|  | XP\_065058319\_14272-model24 | -22.0941 |
| # cluster 25 - Size: 1 |  |  |
|  | XP\_065058319\_14272-model25 | -21.9944 |
| # cluster 26 - Size: 1 |  |  |
|  | XP\_065058319\_14272-model26 | -21.9703 |
| # cluster 27 - Size: 1 |  |  |
|  | XP\_065058319\_14272-model27 | -21.8742 |
| # cluster 28 - Size: 2 |  |  |
|  | XP\_065058319\_14272-model28 | -21.5284 |
|  | XP\_065058319\_14272-model28.1 | -20.012 |
| # cluster 29 - Size: 2 |  |  |
|  | XP\_065058319\_14272-model29 | -21.433 |
|  | XP\_065058319\_14272-model29.1 | -19.5286 |
| # cluster 30 - Size: 4 |  |  |
|  | XP\_065058319\_14272-model30 | -21.4127 |
|  | XP\_065058319\_14272-model30.1 | -21.2449 |
|  | XP\_065058319\_14272-model30.2 | -20.6219 |
|  | XP\_065058319\_14272-model30.3 | -19.9136 |
| # cluster 31 - Size: 1 |  |  |
|  | XP\_065058319\_14272-model31 | -20.8818 |
| # cluster 32 - Size: 2 |  |  |
|  | XP\_065058319\_14272-model32 | -20.6596 |
|  | XP\_065058319\_14272-model32.1 | -19.5679 |
| # cluster 33 - Size: 3 |  |  |
|  | XP\_065058319\_14272-model33 | -20.3838 |
|  | XP\_065058319\_14272-model33.1 | -20.2586 |
|  | XP\_065058319\_14272-model33.2 | -19.6961 |
| # cluster 34 - Size: 2 |  |  |
|  | XP\_065058319\_14272-model34 | -20.3033 |
|  | XP\_065058319\_14272-model34.1 | -15.92 |
| # cluster 35 - Size: 1 |  |  |
|  | XP\_065058319\_14272-model35 | -20.0107 |
| # cluster 36 - Size: 1 |  |  |
|  | XP\_065058319\_14272-model36 | -19.8704 |
| # cluster 37 - Size: 1 |  |  |
|  | XP\_065058319\_14272-model37 | -19.7974 |
| # cluster 38 - Size: 1 |  |  |
|  | XP\_065058319\_14272-model38 | -19.6672 |
| # cluster 39 - Size: 1 |  |  |
|  | XP\_065058319\_14272-model39 | -18.7525 |
| # cluster 40 - Size: 1 |  |  |
|  | XP\_065058319\_14272-model40 | -18.5321 |
| # cluster 41 - Size: 1 |  |  |
|  | XP\_065058319\_14272-model41 | -18.3398 |
| # cluster 42 - Size: 2 |  |  |
|  | XP\_065058319\_14272-model42 | -17.8992 |
|  | XP\_065058319\_14272-model42.1 | -14.6785 |
| # cluster 43 - Size: 1 |  |  |
|  | XP\_065058319\_14272-model43 | -17.738 |
| # cluster 44 - Size: 1 |  |  |
|  | XP\_065058319\_14272-model44 | -17.0265 |
| # cluster 45 - Size: 2 |  |  |
|  | XP\_065058319\_14272-model45 | -16.9273 |
|  | XP\_065058319\_14272-model45.1 | -16.2799 |
| # cluster 46 - Size: 1 |  |  |
|  | XP\_065058319\_14272-model46 | -16.734 |
| # cluster 47 - Size: 1 |  |  |
|  | XP\_065058319\_14272-model47 | -16.296 |
| # cluster 48 - Size: 1 |  |  |
|  | XP\_065058319\_14272-model48 | -16.2227 |
| # cluster 49 - Size: 1 |  |  |
|  | XP\_065058319\_14272-model49 | -16.0935 |
| # cluster 50 - Size: 1 |  |  |
|  | XP\_065058319\_14272-model50 | -15.6162 |
| # cluster 51 - Size: 1 |  |  |
|  | XP\_065058319\_14272-model51 | -14.8854 |
|  |  |  |
